# Single frequency intake of α-linolenic acid rich phytosterol esters attenuates atherosclerosis risk factors in hamsters fed a high fat diet

**DOI:** 10.1186/s12944-016-0185-8

**Published:** 2016-02-03

**Authors:** Qianchun Deng, Xiao Yu, Jiqu Xu, Xiuying Kou, Mingming Zheng, Fenghong Huang, Qingde Huang, Lan Wang

**Affiliations:** Oil Crops Research Institute, Chinese Academy of Agricultural Sciences, 2 Xudong Second Road, Wuhan, 430062 P. R. China; Hubei Key Laboratory of Lipid Chemistry and Nutrition, Wuhan, 430062 China; Functional Oil Laboratory Associated by Oil Crops Research Institute, Chinese Academy of Agricultural Sciences and Infinite (China) Co., LTD, Guangzhou, 510623 China; Institute for Farm Products Processing and Nuclear-Agricultural Technology, Hubei Academy of Agricultural Science, Wuhan, 430064 China

**Keywords:** α-linolenic acid rich phytosterol esters, Dose and frequency, Lipid profile, Intestinal sterol transporters, α-linolenic acid metabolism

## Abstract

**Background:**

Emerging evidence suggested phytosterol esters (PE) exhibited an advantage over naturally occurring phytosterols in reducing atherosclerosis risk factors due to improved fat solubility and compatibility. However, the effects of dietary patterns of PE on lipid-lowering activity were limited and inconsistent. This study aimed to explore the effects of dose and frequency of α-linolenic acid rich phytosterol esters (ALA-PE) on cholesterol and triglyceride metabolism markers focused on intestinal cholesterol absorption and bioconversion of ALA in liver.

**Methods:**

**Dose-dependency study** Male Syrian golden hamsters were fed high-fat diets (HFD) containing low, medium and high dose of ALA-PE (0.72 %, 2.13 % and 6.39 %) for 6 weeks. The high fat diet contained 89.5 % chow diet, 0.2 % cholesterol, 10 % lard and 0.3 % bile salt.

**Dose-frequency study** Male Syrian golden hamsters were provided: (I) 0.4 mL/100 g peanut oil by gavage once a day; (II) 0.4 mL/100 g ALA-PE by gavage once a day; (III) 0.2 mL/100 g ALA-PE by gavage twice a day; (IV) 0.133 mL/100 g ALA-PE by gavage three times a day; (V) 0.1 mL/100 g ALA-PE by gavage four times a day for 6 weeks with a high-fat diet simultaneously.

**Results:**

ALA-PE dose-dependently lowered plasma total cholesterol (TC), triglyceride (TG) and low-density lipoprotein cholesterol (LDL-C) concentrations with a maximal decrease of 42 %, 59 % and 73 %, respectively (*p* < 0.05). Compared to HFD, TC, LDL-C and TG concentrations were significantly lower (*p* < 0.01) in hamsters consumed HFD plus ALA-PE for 1–4 times per day but there were not remarkable differences among different consumption frequencies. No significant changes in plasma antioxidant capacity and lipid peroxidation levels were observed among HFD and HFD plus different doses of ALA-PE groups. The contents of hepatic α-linolenic (ALA), docosapentaenoic (DPA) and docosahexaenoic (DHA) acids were dose-dependently increased in different ALA-PE groups compared to those in HFD group. The abundance of mRNA for intestinal sterol transporters Niemann-Pick C1-Like 1 (NPC1L1), ATP-binding cassette (ABC) transporters ABCG5 and ABCG8 indicated no significant differences among all groups.

**Conclusion:**

ALA-PE dose-dependently improved lipid profile in hamsters fed HFD independent of intestinal ABCG5, ABCG8 and NPC1L1, accompanying by increased conversion of ALA to DPA and DHA in liver. ALA-PE manifested “once a day” lipid-lowering efficacy, highlighting a promising preventive strategy for metabolic syndrome.

## Background

Although the declining trend in cardiovascular diseases (CVDs) mortality has been observed in high-income countries, CVDs are still the leading causes of noncommunicable disease (NCD) deaths. Approximately 31 % of global deaths were attributable to CVDs and most CVD deaths occur in developing countries as reported in 2012 according to data from the WHO. Elevated levels of total cholesterol (TC), especially low-density lipoprotein cholesterol (LDL-C), and triglyceride (TG) are major causes of atherosclerosis and subsequent CVDs [1, 2]. Therefore, it is imperative to explore the specific early intervention strategies for CVD prevention and control.

Increasing evidence demonstrated that dietary modifications including diets rich in phytochemicals reduced one or more risk factors of CVDs [3]. Phytosterols, as a naturally occurring phytonutrient, are well-known as an adjunct to pharmacologic therapy for the ability of inhibiting intestinal cholesterol absorption, thereby reducing plasma cholesterol levels [4]. However, it was not yet clear whether the sterols are taken up by enterocytes through energy-independent passive diffusion or intestinal sterol transporters mediated process [5]. Furthermore, the mechanism of action of phytosterols was still enigmatic and being investigated. Currently, there were three proposed effects based on cholesterol absorption stages, including competitive solubilization and co-crystallization at gastric duodenal levels, decreased hydrolysis of cholesterol esters in luminal micelle and inhibited cholesterol transport from intestinal lumen to lymph [6]. High doses of phytosterols intake were needed to achieve maximal reductions of cholesterol levels due to the weak solubility in both water and fat. Esterification of phytosterols with fatty acids has been suggested for improved compatibility and potential of producing delivery systems for both phytosterols and esterified fatty acids [7, 8]. N-3 polyunsaturated fatty acids (n-3 PUFAs) consumption was intimately associated with reduced cardiovascular risk [9]. N-3 PUFAs rich phytosterol esters were proved to evidently alleviate hypercholesterolemia [10]. Within the n-3 series, α-linolenic acid (ALA), as the precursors of eicosapentaenoic (EPA), docosapentaenoic (DPA) and docosahexaenoic (DHA) acids, may share some of the physiological and functional attributes of n-3 long-chain PUFAs [11]. A recent study revealed that ALA rich phytosterol esters showed improved effect on lipid profile compared to EPA-DHA rich phytosterol esters [12]. Therefore, the potential therapeutic advantage of phytosterols esterified with ALA should be considered in order to maximize the lipid-lowering activity.

Previous studies highlighted the influence of structure variations at lateral chain and rings (oxyphytosterols) on the absorption efficiency of phytosterols and subsequent intestinal cholesterol metabolism [13]. More importantly, the cholesterol-lowering efficacy of phytosterols was significantly affected by dietary background and intake modalities [14–17]. Nevertheless, it remained elusive whether the esterified ALA in the lateral chain affected the absorption and subsequent dose and frequency response of phytosterols on lipid-lowering properties in a complementary or synergistic way. On the basis of the biocatalyst prepared ALA rich phytosterol esters, we investigated the effects of consumption dose and frequency of ALA rich phytosterol esters on lipid-lowering activity. Furthermore, the gene expression involved in regulating intestinal sterol absorption, and the conversion of ALA to n-3 long-chain PUFAs were conducted to identify the underlying mechanisms that produce them.

## Results

### Body weight and food intake

As shown in Table 1, low, medium and high dose of ALA-PE exhibited no significant inhibitory effect on body weight gain after 6 weeks of high-fat feeding. The weekly food intake among all groups also revealed no significant differences throughout the whole experimental period.

**Table 1:** Effect of α-linolenic acid rich phytosterol esters on body weight and food intake of high fat fed hamsters

Groups	Initial weight(g)	Final weight(g)	Food intake(g/day)
HFD	98.6 ± 3.3	117.3 ± 3.1	7.14 ± 1.26
L-ALA-PE	96.8 ± 4.7	121.5 ± 3.3	7.56 ± 2.55
M- ALA-PE	97.8 ± 2.8	117.6 ± 2.7	8.12 ± 2.34
H- ALA-PE	98.2 ± 3.8	118.7 ± 2.6	7.35 ± 2.31

HFD: High fat control group

L-ALA-PE: Low dose of α-linolenic acid rich phytosterol esters group

M-ALA-PE: Medium dose of α-linolenic acid rich phytosterol esters group

H-ALA-PE: High dose of α-linolenic acid rich phytosterol esters group

### Plasma and hepatic lipid profiles

#### Dose dependency of α-linolenic acid rich phytosterol esters supplementation

**Fig. 1:**
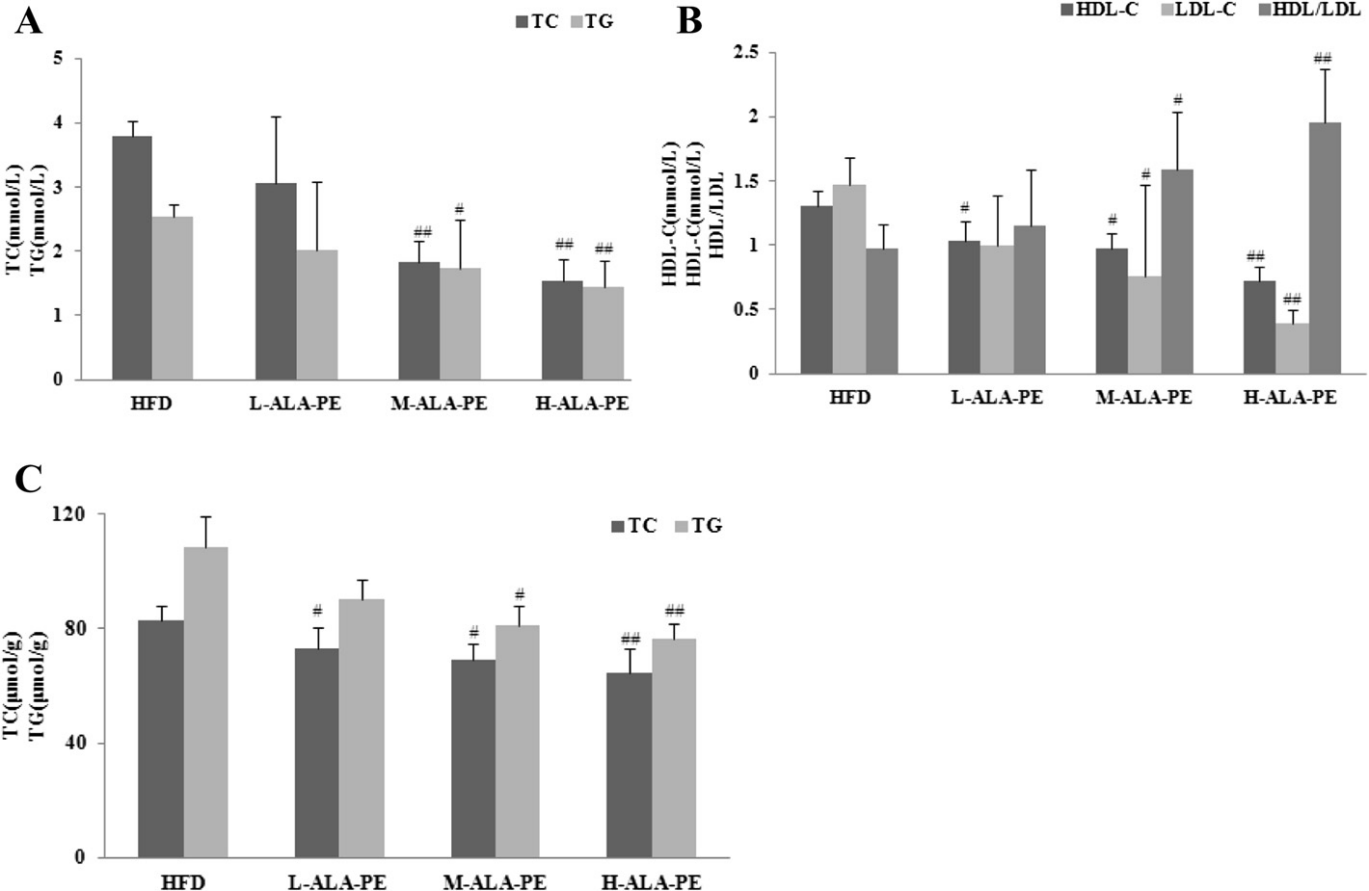
Effect of dose–response of α-linolenic acid rich phytosterol esters on plasma lipid levels of high fat fed hamsters. **a** The plasma TC and TG. **b** The plasma HDL-C, LDL-C and LDL/HDL. **c** The hepatic TC and TG. ^#^ *p* < 0.05 vs. HFD, ^##^ *p* < 0.01 vs. HFD; HFD: High fat control group; L-ALA-PE: Low dose of α-linolenic acid rich phytosterol esters group; M-ALA-PE: Medium dose of α-linolenic acid rich phytosterol esters group; H-ALA-PE: High dose of α-linolenic acid rich phytosterol esters group

As shown in Fig. 1, low dose of ALA-PE partially reduced plasma TC, LDL-C and TG levels in hamsters fed HFD, while middle and high doses of ALA-PE significantly ameliorated HFD-induced increase of plasma TC, LDL-C and TG levels (*p* < 0.05). Additionally, different doses of ALA-PE continuously lowered plasma HDL-C levels in hamsters fed HFD. Moreover, ALA-PE supplementation decreased hepatic TC and TG concentrations in HFD-fed hamsters in a dose-dependent manner, revealing significant differences between HFD and middle, high doses of ALA-PE treated groups (*p* < 0.05).

#### Dose frequency of α-linolenic acid rich phytosterol esters supplementation

**Fig. 2:**
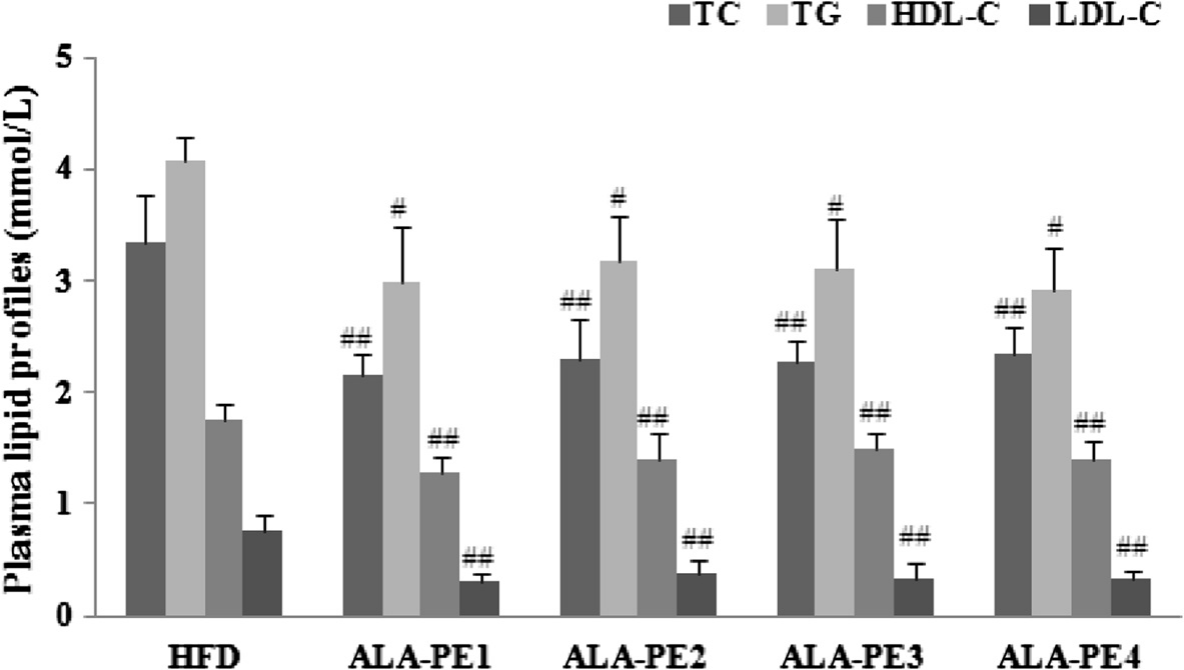
Effect of dose-frequency of α-linolenic acid rich phytosterol esters on plasma lipid levels of high fat fed hamsters. ^#^ *p* < 0.05 vs. HFD, ^##^ *p* < 0.01 vs. HFD; HFD: High fat control group; ALA-PE1: α-linolenic acid rich phytosterol esters group1, received α-linolenic acid rich phytosterol esters by gavage once a day; ALA-PE2: α-linolenic acid rich phytosterol esters group2, received α-linolenic acid rich phytosterol esters by gavage twice a day; ALA-PE3: α-linolenic acid rich phytosterol esters group3, received α-linolenic acid rich phytosterol esters by gavage three times a day; ALA-PE4: α-linolenic acid rich phytosterol esters group4, received α-linolenic acid rich phytosterol esters by gavage four times a day

The data in Fig. 2 showed that based on the same administration dosage, intake frequency and time had no effects on the lipid-lowering activity of ALA-PE. And significant decrease in plasma TC (*p* < 0.01), TG (*p* < 0.05), LDL-C levels (*p* < 0.01), as well as HDL-C (*p* < 0.01), were observed in hamsters after different dose frequency of ALA-PE supplementation.

### Plasma antioxidant capacity and lipid peroxidation

**Fig. 3:**
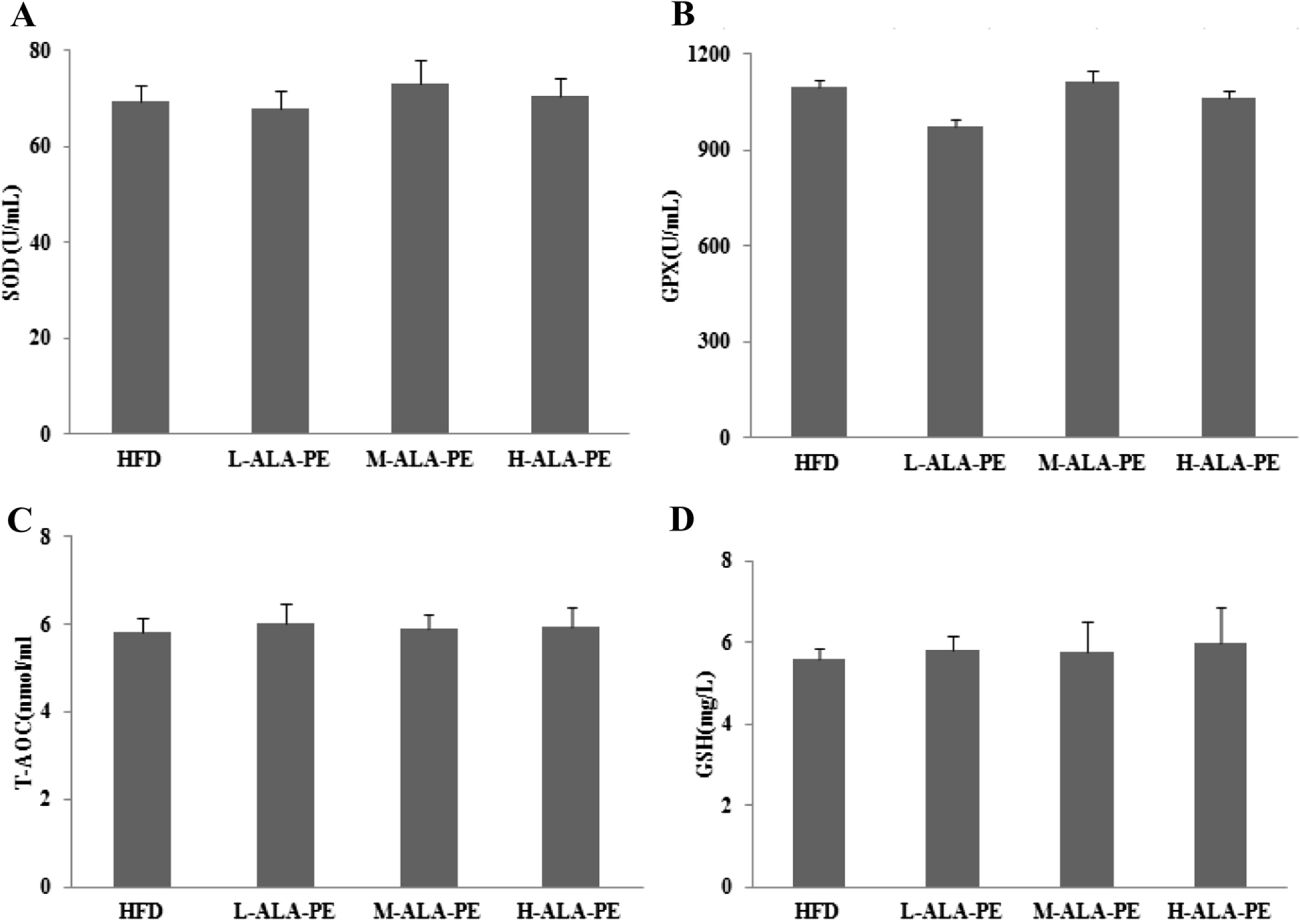
Effect of α-linolenic acid rich phytosterol esters on plasma antioxidant capacity and lipid peroxidation of high fat fed hamsters. **a** Plasma SOD (**b**) Plasma GPX (**c**) Plasma T-AOC (**d**) Plasma GSH. ^#^ *p* < 0.05 vs. HFD, ^##^ *p* < 0.01 vs. HFD; HFD: High fat control group; L-ALA-PE: Low dose of α-linolenic acid rich phytosterol esters group; M-ALA-PE: Medium dose of α-linolenic acid rich phytosterol esters group; H-ALA-PE: High dose of α-linolenic acid rich phytosterol esters group

Interestingly, no significant difference was observed in SOD, GSH, GPX activities and T-AOC level between HFD and HFD plus different doses of ALA-PE groups (Fig. 3).

### Hepatic ALA, DPA and DHA levels

**Fig. 4:**
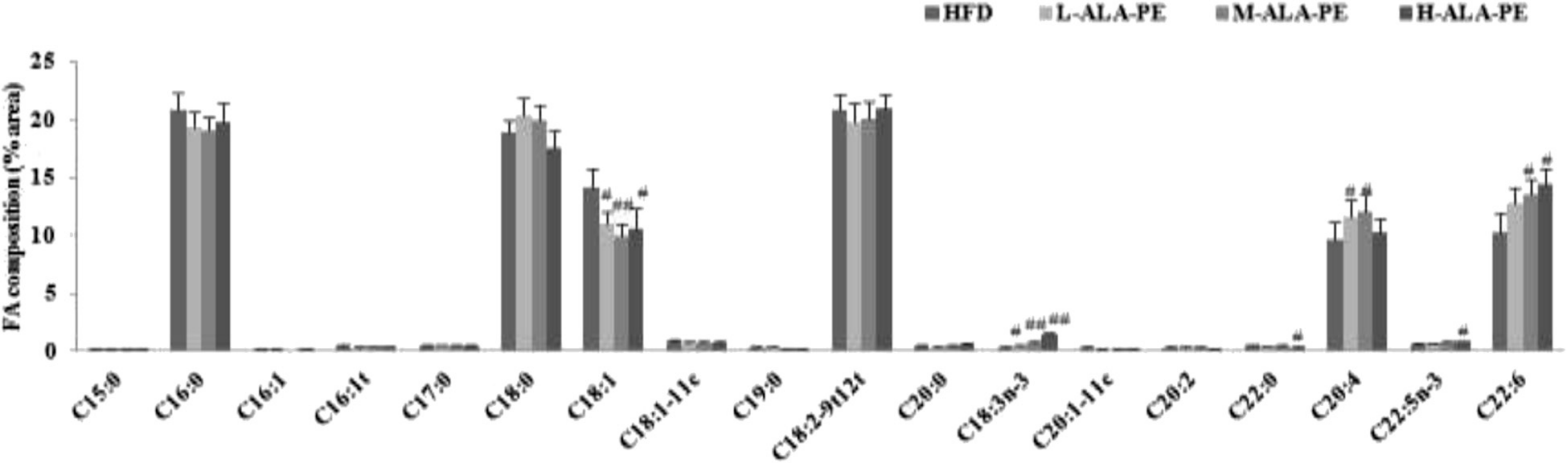
Effect of α-linolenic acid rich phytosterol esters on hepatic fatty acid composition of high fat fed hamsters. ^#^ *p* < 0.05 vs. HFD, ^##^ *p* < 0.01 vs. HFD; HFD: High fat control group; L-ALA-PE: Low dose of α-linolenic acid rich phytosterol esters group; M-ALA-PE: Medium dose of α-linolenic acid rich phytosterol esters group; H-ALA-PE: High dose of α-linolenic acid rich phytosterol esters group

As manifested in Fig. 4, we found that ALA-PE significantly increased the contents of hepatic ALA, DPA and DHA in HFD-fed hamsters with a dose–response relationship. The contents of ALA increased by 82 % (*p* < 0.05), 168 % (*p* < 0.01) and 421 % (*p* < 0.01), concentrations of DPA increased by 16 %, 33 % and 47 % (*p* < 0.05), and DHA levels increased by 24 %, 31 % (*p* < 0.05) and 40 % (*p* < 0.05), respectively, when compared with the HFD group.

### Intestinal NPC1L1, ABCG5 and ABCG8 mRNA levels

**Fig. 5:**
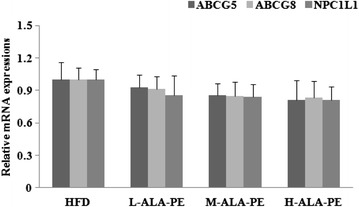
Effect of α-linolenic acid rich phytosterol esters on mRNA levels of intestinal ABCG5, ABCG8 and NPC1L1 of high fat fed hamsters. ^#^ *p* < 0.05 vs. HFD, ^##^ *p* < 0.01 vs. HFD; HFD: High fat control group; L-ALA-PE: Low dose of α-linolenic acid rich phytosterol esters group; M-ALA-PE: Medium dose of α-linolenic acid rich phytosterol esters group; H-ALA-PE: High dose of α-linolenic acid rich phytosterol esters group

As depicted in Fig. 5, different doses of ALA-PE exerted no significant effects on the mRNA expressions of intestinal sterol transporters NPC1L1, ABCG5 and ABCG8.

## Discussion

### The dose and frequency responses of α-linolenic acid rich phytosterol esters supplementation

A dose–response relationship was exhibited between α-linolenic acid rich phytosterol esters dose and plasma lipid levels in our study. The results revealed that 0.72–6.39 % phytosterol esters in HFD, approximately equivalent to 2.4–8.9 g/d for people, continuously reduced plasma TC, TG and LDL-C levels, as well as the hepatic TC and TG concentrations. Similar dose-dependent cholesterol-lowering effect of phytosterols-enriched margarine was also observed in both statin users and non-users [18]. Based on the mechanism of action of phytosterols, a continuous supply of phytosterol esters was needed to interact with and facilitate the excretion of gut cholesterol and bile acids. However, our data indicated that comparable lipid-lowering effect was revealed among the different consumption frequencies of phytosterol esters (1–4 times/day at daily consumption of 0.4 mL/100 g b.w), which agreed with findings from a randomized, double-blind crossover study [19]. While the other study reported that multiple smaller doses but not one large dose of physterols showed greater cholesterol-lowering effect [20]. Considering the differences in cholesterol-lowering efficacy between phytosterol esters and free phytosterols induced by frequency of intake, the fatty acids delivered by phytosterol esters may increase the gastrointestinal responses to phytosterols similar to meal intake, allowing phytosterol esters to continuously exhibit their effect on inhibiting cholesterol absorption independent of the intake occasion [15]. Furthermore, the length of experimental period may affect the frequency-response of phytosterol esters to manifest itself.

### The molecular mechanisms of α-linolenic acid rich phytosterol esters supplementation

Triglycerides are considered to be an independent risk factor for CVD. Recent studies reported that combination of phytosterols and n-3 PUFA reduced CVD risk in a complementary and synergistic way [21]. Our study revealed that α-linolenic acid rich phytosterol esters consumption was associated with dose-dependently reduced plasma and hepatic triacylglycerol levels. The TG-lowering effect of phytosterol esters mainly attributed to the esterified α-linolenic acid in side chain. Furthermore, the expanded evidence of α-linolenic acid for TG-lowering effect was intimately associated with its conversion to EPA, DPA and DHA, which had a higher efficacy in modulating physiological responses [11]. Our analyses demonstrated that significantly increased DPA and DHA levels were incorporated into the hepatic lipid pools of hamsters fed α-linolenic acid rich phytosterol esters. Mechanically, α-linolenic acid exerted most of its effects by modulating lipoprotein (a) and apolipoproteins A-1 and B. EPA and DHA exerted most of its TG-lowering effects by reducing TG synthesis through regulating the hepatic expression of sterol regulatory element-binding protein-1 (SREBP-1). Furthermore, long chain n-3 PUFA up-regulated the expression of peroxisomal acyl-CoA oxidase, carnitine palmitoyl transferase 1 (CPT1) and mitochondrial uncoupling proteins, which facilitated fatty acid β-oxidation in liver mitochondria [9].

N-3 PUFA rich phytosterol esters are now well-known dietary adjuncts that effectively lower cholesterol levels [12]. As shown in this study, significant and dose-dependent cholesterol-lowering effects of α-linolenic acid rich phytosterol esters were revealed in hamster fed HFD. The hydrolysis of phytosterol esters and the presence of free phytosterols were proved to be necessary to induce an optimum cholesterol-lowering effect [22]. However, the exact mechanism of action of phytosterols remained to be clearly understood. Although the displacement of cholesterol from mixed micelles within the intestinal lumen seemed to be an important mechanism of action of phytosterols [6]. Recent researches revealed that the sterol transporters Niemann-Pick C1-Like 1 (NPC1L1), ABCG5 and ABCG8 were critical in regulating cholesterol flux at the intestinal enterocyte level [23]. Therefore, it is reasonable to speculate that phytosterols may decrease cholesterol absorption through regulating the expression of intestinal NPC1L1, ABCG5 and ABCG8. However, the results in current study do not support this hypothesis. α-linolenic acid rich phytosterol esters exerted no significant effect on the mRNA expressions of NPC1L1, ABCG5 and ABCG8 in intestine, indicating that α-linolenic acid phytosterol esters decreased plasma cholesterol independently of the gene expression of the influx and efflux transporters. Our results were consistent with preliminary studies conducted in hamsters supplemented with stanol esters [24, 25]. Of course, these findings could not exclude the possible changes in protein expression or activity of these sterol transporters through posttranscriptional regulation by phytosterol esters. In addition, other alternative mechanisms might be involved in decreased plasma cholesterol concentration by α-linolenic acid rich phytosterol esters, including the regulation of endogenous cholesterol synthesis through inhibition of 3-hydroxy-3-methylglutaryl-CoA reductase (HMGR), or activation of the liver X receptor (LXR) target pathways [26].

In all, these compounds act on multiple targets, phytosterols have long been recognized for their cholesterol-lowering action with little effects on triacylglycerol [27, 28]. However, one recent research has highlighted the complementary plasma triglyceride-lowering responses to phytosterols, which was associated with decreased hepatic VLDL secretion [29]. Moreover, in response to phytosterol esters supplement, hamsters manifested a lower plasma HDL-C levels in a dose-dependent manner than those in high-fat diet fed animals, which indicated that the cholesterol-lowering effect of phytosterol esters was partly due to promoting the selective uptake of cholesteryl ester from HDL mediated by increased protein expressions of SR-BI, the first molecularly well-defined and physiologically relevant HDL receptor [30, 31].

## Conclusion

α-linolenic acid rich phytosterol esters dose-dependently attenuated HFD induced abnormal lipid metabolism independent of the expression of intestinal sterol transporters NPC1L1, ABCG5 and ABCG8, paralleling with increased DPA and DHA levels synthesized from α-linolenic acid. Moreover, α-linolenic acid rich phytosterol esters manifested“once a day efficacy” on reductions of serum lipid levels during 6 weeks of consumption.

## Methods

### Chemicals and materials

α- linolenic acid rich phytosterols esters was prepared by enzymatic catalysis in our laboratory.

### Animal treatment

Male Syrian golden hamsters weighing 90–100 g were obtained from Wuhan Institute of Biological Products Co., Ltd. The animals were housed in stainless steel cages at a controlled room temperature (22 ± 2 °C) and a relative humidity (65 %–75 %) with a 12:12 light dark cycle. The animals were taken care of according to the Guiding Principles in the Care and Use of Laboratory Animals published by the US National Institutes of Health.

### Dose-dependency study

After one week of acclimatization with chow diet, the animals were randomly divided into 5 groups of 10 animals in each with high fat diet for 6 weeks as the following: (I) High fat control group (HFD), received 93.61 % high fat diet and 6.39 % corn starch; (II) Low dose of α-linolenic acid rich phytosterol esters group (L-ALA-PE), received 93.61 % high fat diet, 0.72 % α-linolenic acid rich phytosterol esters and 5.67 % corn starch; (III) Medium dose of α-linolenic acid rich phytosterol esters group (M-ALA-PE), received 93.61 % high fat diet, 2.13 % α-linolenic acid rich phytosterol esters and 4.26 % corn starch; (IV) High dose of α-linolenic acid rich phytosterols esters group (H-ALA-PE), received 93.61 % high fat diet and 6.39 % α-linolenic acid rich phytosterol esters. The high fat diet contained 89.5 % chow diet, 0.2 % cholesterol, 10 % lard and 0.3 % bile salt.

### Dose-frequency study

After one week of acclimatization with chow diet, the animals were randomly divided into 5 groups of 10 animals in each with high fat diet for 6 weeks as the following: (I) High fat control group (HFD), administered 0.4 mL/100 g of peanut oil by gavage once a day at 9 AM; (II) α-linolenic acid rich phytosterol esters group 1 (ALA-PE1), received 0.4 mL/100 g α-linolenic acid rich phytosterol esters by gavage once a day at 9 AM; (III) α-linolenic acid rich phytosterol esters group 2 (ALA-PE2), received 0.2 mL/100 g α-linolenic acid rich phytosterol esters by gavage twice a day at 9 AM and 5 PM, respectively; (IV) α-linolenic acid rich phytosterol esters group 3 (ALA-PE3), received 0.133 mL/100 g α-linolenic acid rich phytosterol esters by gavage three times a day at 9 AM, 1 PM and 5 PM, respectively; (V) α-linolenic acid rich phytosterol esters group 4 (ALA-PE4), received 0.1 mL/100 g α-linolenic acid rich phytosterol esters by gavage four times a day at 9 AM, 11:30 AM 2:30 PM and 5 PM, respectively.

Feed and water were changed every day, and the body weights of the hamsters were recorded once per week. Daily feed (g) was calculated on a per rat daily basis. At the end of the experiment, the hamsters were sacrificed after an overnight fasting. Plasma was collected from blood by centrifuge at 3500 g for 10 min, and then stored at −80 °C. Fresh liver and small intestine samples were quickly frozen by liquid nitrogen and stored at–80 °C for further analysis.

### Analysis of plasma and hepatic lipid parameters

Plasma TC, TG, LDL-C and HDL-C concentrations were determined by enzymatic colorimetric methods using commercial kits (Biosino Bio-Technology and Science Inc., Beijing, China). Hepatic lipids were extracted as previously described by Folch et al. (1957) [32]. Briefly, approximately 100 mg of liver tissue was homogenized in chloroform/methanol (2/1, v/v) to a final volume of 20 times the tissue sample. The homogenate was filtered with Whatman No.1 filter paper to obtain the liquid phase. The extract was then dried under N_2_ and resuspended in isopropanol. Hepatic TC and TG levels were measured using the same enzymatic commercial kits as the plasma analysis.

### Analysis of plasma antioxidant capacity

The T-AOC level was determined using the Fe^3+^ reduction method according to the commercial test kits (Nanjing Jiancheng Bioengineering Institute, Nanjing, China). The SOD activity was estimated basing on the xanthine-xanthine oxidase-nitrite method according to Fridovich with slight modification [33]. The GSH content was estimated by the use of commercial kit (Nanjing Jiancheng Bioengineering Institute, China) based on the method of Eady et al. [34]. GPX activity was determined by the use of commercial kit (Nanjing Jiancheng Bioengineering Institute, China) based on the method of Sazuka et al. [35].

### Analysis of hepatic fatty acid composition

Fatty acids were transmethylated based on the method of Bicalho et al. [36]. In brief, the total lipid extracts in chloroform were evaporated to dryness under a stream of N_2_ at 40 °C. And 500 μL of freshly prepared 5 % acetylchloride (Sigma–Aldrich) in methanol (HPLC grade, Merck KGaA, Darmstadt) was added to each tube. The tubes were tightly capped and heated for 30 min at 90 °C. After cooling, 200 μL of n-hexane (HPLC grade, Merck KGaA, Darmstadt) was added and the tubes were shaken for 1 min. N-hexane containing fatty acid methyl esters was directly taken for gas chromatographic analysis. Fatty acid methyl esters were separated using Agilent 6890 GC with an HP-88 fused silica capillary column (100 m × 0.25 mm, ID × 0.20 μm thickness, J&W 112-88A7, Agilent Technologies) and a flame ionization detector. The oven temperature started at 50 °C and held for 1 min, increased to 175 °C at 15 °C/min, and then increased to 250 °C at 1 °C/min. The injector temperature and detector temperature were set at 250 °C. Helium was used as a carrier gas (1.5 mL/min), and the injection volume was 1 μL in a splitless mode. The fatty acid methyl esters were identified by comparing with authentic standards (GL-463, Nu-Chek Prep) and their relative concentrations (mol % of total lipids) were calculated by the percentage area method with proper normalization.

### Real-time quantitative polymerase chain reaction (PCR) analysis

Total RNA was extracted from small intestine tissue using the TRIzol reagent (Ambion®, life technologies, USA) according to the manufacturer’s instructions. Messenger RNA (mRNA) expressions of the target genes were quantified by quantitative reverse transcriptase (qRT)-PCR using the SYBR green-based kit (TaKaRa BIO Inc., Dalian) with specific primers using an RT-PCR machine (7900HT; Applied Biosystems, Forster, CA, USA). The mRNA level of 18S rRNA was quantified as an endogenous control, and results were calculated by a comparative 2^−ΔΔCt^ method. Primer sequences were as follows: ABCG5 (AF312713), 5’- TGATTGGCAGCTATAATTTTGGG-3’ and 5’- GTTGGGCTGCGATGGAAA-3’; ABCG8 (AF324495), 5’- TGCTGGCCATCATAGGGAG-3’ and 5’- TCCTGATTTCATCTTGCCACC-3’; NPC1L1 (AY437866), 5’- CCTGACCTTTATAGAACTCACCACAGA-3’ and 5’- GGGCCAAAATGCTCGTCAT-3’; 18S rRNA (M33069), 5’- TAAGTCCCTGCCCTTTGTACACA-3’and 5’- GATCCGAGGGCCTCACTAAAC-3’.

### Statistical analysis

All data were presented as the mean ± SD. The data were analyzed using one-way ANOVA followed by Tukey’s test to evaluate differences between groups using SPSS statistical software. Differences among groups were considered significant at *p* < *0.05.*

### Ethical approval

The animals used in our study were taken care of according to the Guiding Principles in the Care and Use of Laboratory Animals published by the U.S. National Institutes of Health. Animal experiments described in our study were approved by the Oil Crops Research Institute Council on Animal Care Committee, Chinese Academy of Agricultural Sciences.

## Abbreviations

ABCG5: ATP-binding cassette transporter G5
ABCG8: ATP-binding cassette transporter G8
ALA: α-linolenic acid
CVDs: cardiovascular diseases
DHA: docosahexaenoic acid
DPA: docosapentaenoic acid
EPA: eicosapentaenoic acid
GSH: glutathione
H-ALA-PE: high dose of α-linolenic acid rich phytosterols esters
HDL-C: high-density lipoprotein cholesterol
HMGR: 3-hydroxy-3-methylglutaryl-CoA reductase
L-ALA-PE: low dose of α-linolenic acid rich phytosterol esters
LDL-C: low-density lipoprotein cholesterol
LXR: liver X receptor
M-ALA-PE: medium dose of α-linolenic acid rich phytosterol esters
MDA: malondialdehyde
n-3 PUFAs: n-3 polyunsaturated fatty acids
NCD: noncommunicable disease
NPC1L1: niemann-Pick C1-Like 1
SOD: superoxide dismutase
T-AOC: total antioxidant capability
TC: total cholesterol
TG: triglyceride
VLDL: very-low-density lipoprotein
